# Prognostic Value of Nevus-Associated Melanoma in Patients with Melanoma

**DOI:** 10.1245/s10434-025-16945-2

**Published:** 2025-02-01

**Authors:** Nazia Riaz, Anne Huibers, Stanley P. Leong, Mohammed Kashani-Sabet, Richard L. White, John T. Vetto, Schlomo Schneebaum, Cristina O’Donoghue, Harrison Howard, Eli Avisar, Jukes P. Namm, Heidi Kosiorek, Barbara Pockaj, Mark Faries, Giorgos Karakousis, Jonathan S. Zager, Roger Olofsson Bagge

**Affiliations:** 1 Department of Surgery, Institute of Clinical Sciences, Sahlgrenska Academy, University of Gothenburg, Gothenburg, Sweden; 2 Department of Surgery, Sahlgrenska University Hospital, Gothenburg, Sweden; 3 Center for Melanoma Research and Treatment, California Pacific Medical Center and Research Institute, San Francisco, CA USA; 4 Department of Surgery, Atrium Health Levine Cancer Center, Charlotte, NC USA; 5 Department of Surgery and Division of Surgical Oncology, Oregon Health and Science University, Portland, OR USA; 6 Department of Surgery, Tel Aviv University, Tel Aviv, Israel; 7 Department of Surgery, Rush University Medical Center, Chicago, IL USA; 8 Department of Surgery, University of South Alabama, Mobile, AL USA; 9 Department of Surgical Oncology, University of Miami School of Medicine, Miami, FL USA; 10 Department of Surgery, Loma Linda University, Loma Linda, CA USA; 11 Department of Quantitative Health Sciences, Mayo Clinic, Scottsdale, AZ USA; 12 Department of General Surgery, Division of Surgical Oncology, Mayo Clinic – Arizona, Phoenix, AZ USA; 13 Department of Surgery, Angeles Clinic and Research Institute, Cedars-Sinai Medical Center, Los Angeles, CA USA; 14 Division of Endocrine and Oncologic Surgery, University of Pennsylvania School of Medicine, Philadelphia, PA USA; 15 Departments of Cutaneous Oncology and Sarcoma, Moffit Cancer Center, Tampa, FL USA

**Keywords:** Cutaneous melanoma, Preexisting nevi, Prognosis, Sentinel lymph node, Recurrent melanoma

## Abstract

**Background:**

Although most melanomas develop de novo, about 30% are nevus-associated melanomas, where the prognostic value is unclear. Our study aimed to determine whether nevus-associated melanoma is associated with sentinel lymph node (SLN) status and prognosis in patients with melanoma.

**Methods:**

The Sentinel Lymph Node Working Group database, which includes comprehensive clinicopathological and outcome data, was utilized to investigate the association of nevus-associated melanoma with SLN status as well as relapse-free (RFS), melanoma-specific (MSS), and overall survival (OS) using multivariable logistic regression and Cox regression modeling.

**Results:**

A total of 3447 adult patients with a median follow-up of 2.6 years (interquartile range 0.9–6.9) were evaluable. Compared with de novo melanomas, nevus-associated melanomas showed a significant correlation with younger age as well as favorable histological features. The presence of a nevus-associated melanoma was not identified as an independent factor for SLN status (odds ratio 1.06, 95% confidence interval [CI] 0.80–1.41; *p *= 0.68). Compared with de novo melanomas, nevus-associated melanomas provided independent prognostic information for a favorable RFS (hazard ratio [HR] 0.67, 95% CI 0.53–0.84; *p *< 0.001), MSS (HR 0.54, 95% CI 0.34–0.85; *p *= 0.008), and OS (HR 0.42, 95% CI 0.30–0.57; *p *< 0.001).

**Conclusion:**

Melanomas associated with pre-existing nevi appear to be an independent favorable prognostic factor for recurrence and survival and may potentially be used as a clinical parameter for identifying patients with lower risk of recurrence.

**Supplementary Information:**

The online version contains supplementary material available at 10.1245/s10434-025-16945-2.

Cutaneous melanoma is an aggressive disease that originates in the melanin-producing skin cells or melanocytes. This disease predominantly affects fair-skinned individuals, specifically of European lineage. The global burden has demonstrated a continued increase in the past several decades. Furthermore, according to the projected estimates, melanoma incidence and mortality is likely to demonstrate an alarming increment of 50% and 68%, respectively, by 2040 [1, 2]. Oncogenic transformation is multifaceted and encompasses a series of complex interactions triggered by ultraviolet radiation in sun-exposed areas. These mostly involve somatic mutations (*BRAF*, *NRAS,* and *KIT*), germline alterations that modify susceptibility (*CDKN2A, MC1R, and BAP1*) and certain phenotypic characteristics such as fair skin type, sun sensitivity, excessive nevus count (>100), or presence of dysplastic nevi [3].

About 70% of cutaneous melanomas develop without any precursor lesion (de novo melanomas), while histological evidence of adjacent nevi characterizes up to 30% of melanomas (nevus- associated melanomas), although a wide range has been reported in earlier studies (18–85%) [4–6]. The notion that melanocytic nevi act as precursor lesions to subsequent melanoma development has been an unresolved issue. This is partly related to the overall low transforming potential of any nevus to melanoma. As such, the lifetime risk for a nevus to transform into a melanoma is 0.03% for men (1:3164) and 0.009% for women (1:10,800) [7]. The exact incidence of nevus-associated melanoma is not known, perhaps due to obliteration of nevi remnants in thicker melanomas; however, a growing body of evidence suggests that nevus-associated melanoma is indeed a biologically and clinically distinct entity. [8–12]

Genetic evaluation studies have shown that melanocytic nevi exhibit a multitude of mutations in the proto-oncogenes involved in the MAP kinase pathway. Among these, ultraviolet (UV)-induced activating mutation in the *BRAF* proto-oncogene (BRAF^V600E/K^), a serine/threonine protein kinase, is found in up to 82% of melanocytic nevi, which heralds the onset of nevi formation and subsequent proliferation, albeit limited [13, 14]. Although regression of melanocytic nevi has been largely attributed to oncogenic-induced senescence, other homeostatic mechanisms have also been proposed [15, 16]. Nevertheless, a linear progression model of melanocytic nevi to melanoma indeed exists, as suggested by the presence of adjacent nevi remnants in a melanoma, possibly attributed to incremental accumulation of mutations [17].

Limited evidence exists regarding the impact of nevus-associated melanoma on oncological outcomes [6]. Given the distinct clinicopathological characteristics, we hypothesized that nevus- associated melanomas are associated with clinical outcomes. The aim of this study was therefore to compare the clinicopathological features of patients diagnosed with nevus-associated melanoma with de novo melanoma. We also sought to determine the prognostic significance of nevus-associated melanoma with regard to sentinel lymph node (SLN) status and survival.

## Methods

### Description of the Patient Cohort

A retrospective analysis of the Sentinel Lymph Node Working Group (SLNWG) database was performed. The SLNWG is an initiative of the Sentinel Node Oncology Foundation and comprises 15 academic medical centers/practices in North America, Europe, and Asia [18]. The SLNWG has assembled a comprehensive database of 12,143 patients diagnosed with stage I–IV melanoma between October 1993 and March 2024. The database includes demographic features, clinicopathological characteristics, treatments, oncological outcomes, and long-term follow-up. Each participating center followed their respective national standards for diagnostic excision, wide local excision, SLN biopsy (SLNB) and pathological evaluation of the specimens, follow-up, and recording of the clinical outcomes.

The inclusion criteria comprised all adult patients ≥ 18 years of age diagnosed with stage I–III melanoma of histological subtypes, including superficial spreading, lentigo maligna, nodular, and acral. Patients with stage IV disease, missing data for pre-existing nevi and melanoma subtype, and those diagnosed with rare histological subtypes of melanoma were excluded. All participating centers submitted prerecorded clinicopathological data (including the presence or absence of pre-existing nevi on pathology slides) to the SLNWG data repository. No additional review of the pathology slides was undertaken for the purpose of this study. Members of the SLNWG obtained ethical approval from their respective institutions for use of the de-identified data for research. The study was performed in accordance with recommendations of the Strengthening the Reporting of Observational Studies in Epidemiology (STROBE) guidelines [19]. Race and ethnicity data were not available from some centers within the database and, as a result, are not presented in the current study.

### Statistical Analyses and Endpoints

SPSS Statistics software version 29.0.2.0 (IBM Corporation, Armonk, NY, USA) was used for statistical analyses. The clinicopathological factors comprised demographic features including age and sex, histological features including edge status, anatomical site (head and neck, trunk, extremities), nevus-associated melanoma (defined as nevi remnants contiguous with melanoma on the pathology specimen) or de nova melanoma, Breslow thickness (mm), ulceration, mitotic rate (per mm^2^), lymphovascular invasion, regression, and SLN status. For descriptive statistics, median and interquartile range (IQR) are reported for continuous parameters, including age and follow-up time (years). Frequency and percentages are reported for categorical variables when comparing the baseline clinicopathological features. For descriptive analyses, Breslow thickness was reported as categories of ≤ 1.0 mm, 1.01–2.0 mm, 2.01–4.0 mm, ≥ 4.0 mm. The Chi-square test was applied for estimating the associations of clinicopathological features among patients with nevus- associated versus de novo melanoma.

The Kaplan–Meier method was applied to investigate the cumulative survival probabilities against the primary endpoint of relapse-free survival (RFS; defined as the time from treatment initiation to melanoma recurrence), and secondary endpoints of melanoma-specific survival (MSS; defined as the time from treatment initiation to death due to melanoma) and overall survival (OS; defined as the time from treatment initiation to death due to any cause). The differences in survival probabilities were estimated using the log-rank test. Multivariable Cox proportional hazards modeling was used to determine the independent prognostic significance of nevus-associated melanoma; hazard ratios (HRs) with corresponding 95% confidence intervals (CIs) are reported. Multivariable logistic regression was applied to assess the independent prognostic value of having a nevus-associated melanoma on SLN status, reporting odds ratios (ORs) with corresponding 95% CIs. A *p*-value < 0.05 was considered significant. In addition to the nevus- associated versus de novo melanomas, the multivariable model included standard clinicopathological variables such as age and Breslow thickness (continuous variables), sex, anatomical sites, edge status, ulceration, regression, mitotic rate, and vascular invasion (categorical variables). The prespecified statistical plan was presented and approved by the SLNWG. Analysis of the data was performed at Sahlgrenska Academy, University of Gothenburg, Gothenburg, Sweden.

## Results

### Clinicopathological Characteristics of the Full Cohort

**Fig. 1:**
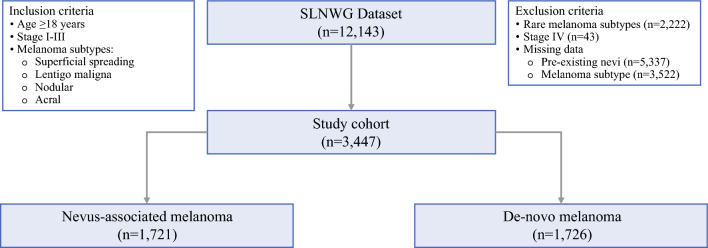
CONSORT diagram summarizing the study cohort. *CONSORT* Consolidated Standards of Reporting Trials, *SLNWG* Sentinel Lymph Node Working Group

The flowchart summarizing the study cohort is shown in Fig. 1. From the original cohort of 12,143 patients, a total of 3447 patients were considered eligible for inclusion in the current study. The median age of the cohort was 62 years (IQR 49.7–72.1) and the median follow-up was 2.6 years (IQR 0.9–6.9). The most common histological subtype was superficial spreading (*n* = 2973, 56.3%) followed by nodular (*n* = 1786, 33.8%), acral (*n* = 260, 4.9%), and lentigo maligna (*n* = 265, 5%). After the initial diagnostic excision, margin status was positive in 43.5% of patients, who then underwent re-excision to achieve a negative margin. SLN status was positive in 22.3% (*n* = 591) of patients, and complete lymph node dissection was performed in 2% (*n* = 62) of patients.

### Correlation of Clinicopathological Characteristics with Nevi-Associated and De Novo Melanomas

A total of 1721 (49.9%) patients were categorized into the nevus-associated melanoma group, while 1726 (50.3%) patients were assigned into the de novo melanoma group. Compared with de novo melanomas, patients with nevus-associated melanomas demonstrated a significant association with younger age, superficial spreading subtype, and favorable histological features such as lower Breslow thickness, non-ulcerated lesions, mitotic rate <1 mm^2^, presence of regression, and clinical stage I disease (*p *< 0.05) (Table 1).

**Table 1:** Association of baseline clinicopathological features of the SLNWG cohort with nevus-associated versus de novo melanomas [*n* = 3447]

Clinicopathological factors	Nevus-associated melanoma [*n* = 1721] (%)	De novo melanoma [*n* = 1726] (%)	*p*-Value
Age, years			<0.001
<62	899 (52.2)	714 (41.4)	
≥62	822 (47.8)	1012 (58.6)	
Missing	0	0	
Sex			0.73
Male	982 (57.1)	1000 (58)	
Female	738 (42.9)	724 (42.0)	
Missing	1 (0.1)	2 (0.1)	
Body site			0.53
Head and neck	280 (16.3)	261 (15.1)	
Extremities	812 (47.2)	808 (46.8)	
Trunk	629 (36.5)	657 (38.1)	
Missing	0	0	
Melanoma subtype			<0.001
Superficial spreading	1131 (65.7)	892 (51.7)	
Nodular	428 (24.9)	670 (38.8)	
Acral	57 (3.3)	76 (4.4)	
Lentigo maligna	105 (6.1)	88 (5.1)	
Missing	0	0	
Breslow thickness, mm			<0.001
≤1.0	475 (27.6)	265 (15.4)	
1.01–2.0	692 (40.2)	632 (36.6)	
2.01–4.0	337 (19.6)	399 (23.1)	
>4.0	215 (12.5)	426 (24.7)	
Missing	2 (0.1)	4 (0.2)	
Ulceration			<0.001
Yes	429 (25)	627 (36.3)	
No	1286 (75)	1091 (63.2)	
Missing	6 (0.3)	8 (0.5)	
Vascular invasion			<0.001
Yes	89 (5.2)	102 (5.9)	
No	1476 (85.8)	1562 (90.5)	
Missing	156 (9.1)	62 (3.6)	
Mitotic rate, mm^2^			<0.001
<1	221 (12.8)	118 (6.8)	
≥1	1110 (64.5)	765 (44.3)	
Missing	390 (22.7)	843 (48.8)	
Regression			<0.001
Yes	261 (15.2)	189 (11)	
No	1066 (61.9)	1384 (80.2)	
Missing	394 (22.9)	153 (8.9)	
Stage			<0.001
I	948 (55.1)	684 (39.6)	
II	437 (25.4)	671 (38.9)	
III	336 (19.5)	371 (21.5)	
Missing	0	0	
Sentinel node status			<0.001
Negative	1381 (80.2)	1262 (73.1)	
Positive	291 (16.9)	300 (17.4)	
Missing	49 (2.8)	164 (9.5)	

*SLNWG* Sentinel Lymph Node Working Group

### Prognostic Impact of Nevus-Associated Melanomas on Sentinel Lymph Node Status

**Fig. 2:**
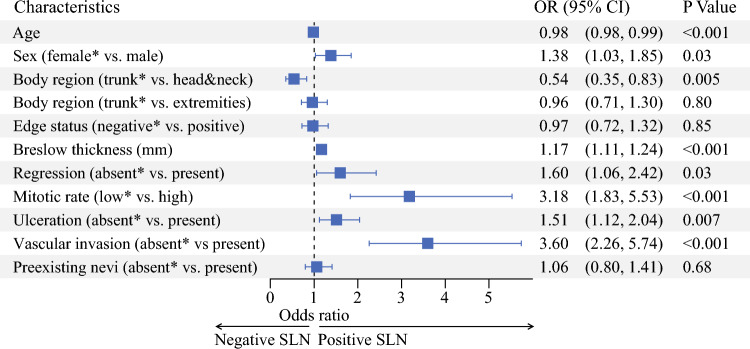
Mutivariable logistic regression analysis: forest plot showing clinicopathological factors associated with sentinel lymph node status (* reference category). *OR* odds ratio, *CI* confidence interval, *SLN* sentinel lymph node

Using multivariable logistic regression analysis, we investigated whether nevus-associated melanoma provided prognostic information regarding SLN status and found that it was not an independent prognostic factor (OR 1.06, 95% CI 0.80–1.41; *p *= 0.68) [Fig. 2].

### Prognostic Significance of Nevi-Associated Melanoma versus De Novo Melanomas

**Fig. 3:**
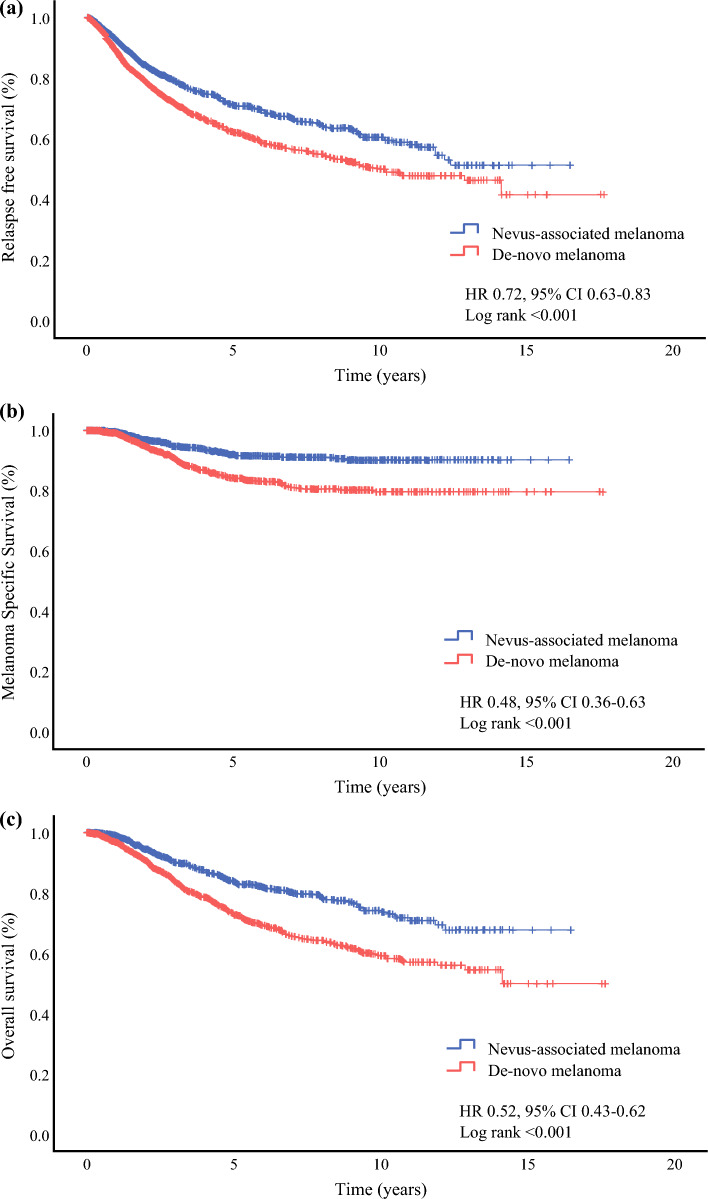
Univariable analyses: Kaplan–Meier curves showing survival probabilities for nevus-associated melanomas versus de novo melanomas. **a** Relapse-free survival; **b** melanoma-specific survival; **c** overall survival. *HR* hazard ratio, *CI* confidence interval

**Fig. 4:**
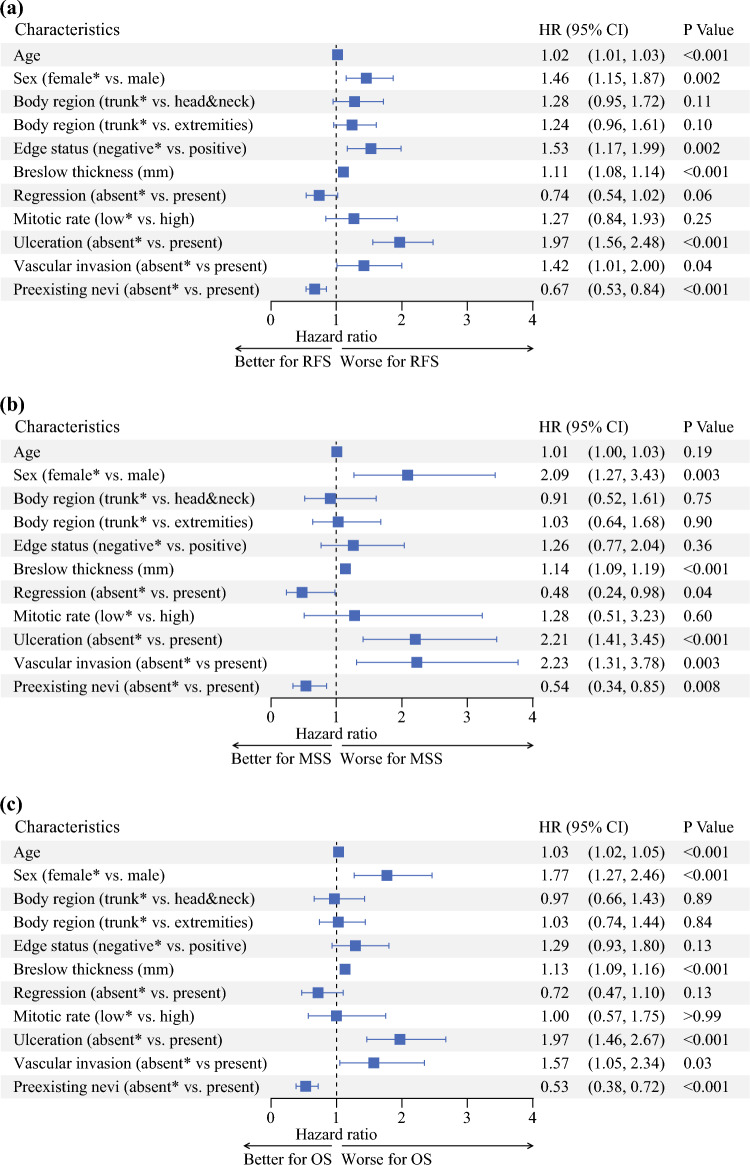
Multivariable Cox regression analysis: forest plots showing the independent prognostic value of nevus-associated melanomas. **a** RFS; **b** MSS; **c** OS (* reference category). *HR* hazard ratio, *CI* confidence interval, *RFS* relapse-free survival, *MSS* melanoma-specific survival, *OS* overall survival

We then evaluated the prognostic significance of nevus-associated melanomas in the full study cohort (*n* = 3447). Univariable analyses showed that compared with patients with de novo melanoma, patients with nevus-associated melanoma experienced a significantly better RFS (HR 0.72, 95% CI 0.63–0.83; *p* < 0.001), MSS (HR 0.48, 95% CI 0.36–0.63; *p* < 0.001), and OS (HR 0.52, 95% CI 0.43–0.62; *p* < 0.001) [Fig. 3]. This was confirmed in multivariable analyses, where nevus-associated melanoma was independently associated with a significantly better RFS (HR 0.67, 95% CI 0.53–0.84; *p* < 0.001), MSS (HR 0.54, 95% CI 0.34–0.85; *p *= 0.008), and OS (HR 0.53, 95% CI 0.38–0.72; *p* < 0.001) [Fig. 4].

### Subgroup Analysis of Nevus-Associated Melanomas in Superficial Spreading Melanomas

Previous studies have demonstrated an association of pre-existing nevi with superficial spreading subtype [4]. We further performed a subgroup analysis evaluating the prognostic relevance of pre-existing nevi in the superficial spreading subtype in the SLNWG cohort. A total of 2023 patients had superficial spreading subtype, among whom histological evidence of pre-existing nevi was present in 56% (*n* = 1131). Compared with de novo melanomas, patients with nevus-associated melanoma experienced a significantly better outcome in univariable analysis for both the primary and secondary endpoints (electronic supplementary material [ESM] Fig. 1). In multivariable analysis, the independent prognosis of nevus-associated melanoma was retained for MSS (HR 0.28, 95% CI 0.12–0.65; *p *= 0.003) and OS (HR 0.52, 95% CI 0.31–0.87; *p *= 0.01), but not for RFS (HR 0.73, 95% CI 0.50–1.07; *p *= 0.11) [ESM Table 1].

## Discussion

In the present study, we examined the significance of having a nevus-associated primary melanoma by analyzing patients in the international multicenter SLNWG database. Following a prespecified hypothesis and analytical strategy, our results showed that compared with de novo melanomas, the presence of a pre-existing nevi is linked with favorable clinicopathological features, and, most importantly, also conveys independent prognostic information for recurrence and survival. Our results also showed that although demonstrating more favorable outcomes, nevus-associated melanomas are not a predictive factor for SLN status.

Increasing evidence suggests that nevus-associated melanomas are characterized by distinct pathophysiological, genetic, and molecular pathways [11, 12, 20]. From a clinical perspective, nevus-associated melanomas are diagnosed at a younger age, exhibit histological characteristics of superficial spreading subtype, and primarily involve the trunk and extremities, anatomic sites that have an intermittent sun exposure. On the other hand, de novo melanomas are mostly diagnosed in older age groups and more frequently demonstrate ulcerative, thicker lesions without evidence of regression [3, 4].

Previous studies investigating the prognostic significance of pre-existing nevi have mostly yielded insignificant associations with clinical outcomes [8, 9, 21–23]. The findings of superior outcomes in nevus-associated melanoma are rather intriguing in this study. Concordant with earlier studies [9], our results showed that nevus-associated melanomas are more likely to be associated with younger age and lower Breslow thickness than de novo melanomas and are predominantly devoid of ulceration and vascular invasion—features that are associated with better prognosis [24]. It is plausible that the association of nevus-associated melanomas with indolent clinicopathological features may have played a role in defining the significant survival advantage in the present study [24]. However, additional investigations are needed to better understand the underlying genetic, epigenetic, environmental, and immunological distinctions that may have contributed to survival advantage in nevus-associated melanomas [11, 12]. For instance, with regard to immunological mechanisms, Schiferle and colleagues, using preclinical models, showed that benign acquired nevus, when transplanted in an immunodeficient patient-derived xenograft model, undergoes spontaneous rejection that was primarily mediated by clonal expansion of nevus resident CD4^+^Th1 cells. Furthermore, their results also showed that nevus-induced augmented adaptive T-cell response may protect against the development of melanoma [25]. These findings point towards a potential therapeutic opportunity whereby the inherent adaptive immunity of melanocytic nevus could be exploited against nevus-associated melanomas. Despite the association with a better prognosis in nevus-associated melanoma, our data did not show any prognostic associations with SLN status. The results remained consistent when an exploratory analysis was performed in a subgroup limited to patients with intermediate-thickness melanomas (results not shown). These findings corroborate with an earlier study [26]. Future studies utilizing spatial transcriptomics and single-cell RNA sequencing will potentially improve our understanding of biological underpinnings between nevus-associated and de novo melanomas [11, 27].

The strength of our study is the utilization of a patient cohort from multiple international centers from two continents with long-term comprehensive oncological follow-up that allowed us to perform a hypothesis-driven prespecified analyses. However, our study has some limitations. First, for the pre-existing nevi category, the histological information for common or dysplastic nevi was not available in the SLNWG database; hence, we were not able to decipher whether the prognostic value would have differed had this information been available. A recent systematic review and meta-analysis of 22 studies showed that up to 56% of melanomas and 71% of in situ melanomas may develop within dysplastic nevi [28]. Nevertheless, accurate prospective evaluation of the associated histological nevus remnant is warranted, not only for improving the understanding of nevi to melanoma progression but also for designing surveillance strategies. Second, it is a matter of speculation whether increasing Breslow thickness could have affected the assignment of melanomas into the de novo category. It has been shown that the proportion of nevus-associated melanomas is higher in thin melanomas and that the increasing tumor thickness may obliterate the pre-existent nevus component [4]. Third, although the missing data and a short median follow-up could have introduced potential bias, we believe that the salient results still remain valid and offer important insights into the prognostic role of pre-existing nevi. Lastly, we acknowledge that the SLNWG database comprises only patients who underwent SLNB and this may have introduced a potential bias influencing our results.

## Conclusion

Our study has reported the clinicopathological and prognostic distinctions between nevus-associated and de novo melanomas. Currently, this distinction does not influence therapeutic decisions; however, future prospective studies with thorough standardized diagnostic evaluation of precursor lesions may potentially guide prognostication, therapeutic decisions and follow-up routines.

## Supplementary Information

Below is the link to the electronic supplementary material.

Supplementary file1 (DOCX 332 KB)

## Data Availability

Requests for access to the de-identified patient data that form the basis of our findings should be directed to the SLNWG (https://snoffoundation.org/slnwg); however, access to the data is at the discretion of the SLNWG.
