# Supplementary material for: Prognostic Value of Nevus-Associated Melanoma in Patients with Melanoma
Source: Ann Surg Oncol. 2025 Feb 1;32(5):3189–97. doi: 10.1245/s10434-025-16945-2 (PMC11976787; doi:10.1245/s10434-025-16945-2)
Supplement: Supplementary file 1 — Supplementary file1 (DOCX 332 KB) [file 10434_2025_16945_MOESM1_ESM.docx]

**Supplementary Figure 1**: Subgroup analyses in the superficial spreading subtype. Kaplan Meir curves showing survival probabilities in nevus associated melanoma versus de novo melanoma groups. A) Relapse free survival B) Melanoma specific survival C) Overall survival.

**Supplementary Table 1:** Multivariable analyses for independent prognostic significance of preexisting nevus in nevus associated melanoma in the superficial spreading subtype in the SLNWG cohort.

| **Clinicopathological features** | **Melanoma Specific Survival** | | **Relapse Free Survival** | | **Overall Survival** | |
| --- | --- | --- | --- | --- | --- | --- |
|  | **95% CI** | **P-value** |  | **P-value** | **95% CI** | **P- value** |
| Age (years) | 1.00 (0.97-1.03) | >0.99 | 1.03 (1.01-1.04) | <0.001 | 1.05 (1.03-1.07) | <0.001 |
| Gender (female vs male) | 1.55 (0.62-3.84) | 0.35 | 1.19 (0.79-1.78) | 0.41 | 1.14 (0.67-1.94) | 0.64 |
| Anatomical site (trunk vs head & neck) | 0.51 (0.20-1.34) | 0.17 | 1.67 (1.03-2.72) | 0.04 | 1.10 (0.58-2.11) | 0.77 |
| Anatomical site (trunk vs extremities) | 0.50 (0.19-1.34) | 0.17 | 1.02 (0.66-1.58) | 0.92 | 0.66 (0.38-1.15) | 0.14 |
| Edge status (negative vs positive) | 1.57 (0.60-4.12) | 0.36 | 1.74 (1.09-2.79) | 0.02 | 1.21 (0.66-2.22) | 0.54 |
| Breslow thickness (mm) | 1.35 (1.23-1.49) | <0.001 | 1.26 (1.18-1.34) | <0.001 | 1.26 (1.17-1.36) | <0.001 |
| Ulceration (absent vs present) | 2.66 (1.15-6.18) | 0.02 | 1.73 (1.12-2.66) | 0.01 | 1.03 (0.58-1.84) | 0.91 |
| Regression (absent vs present) | 0.45 (0.13-1.57) | 0.21 | 1.08 (0.70-1.68) | 0.73 | 1.12 (0.59-2.14) | 0.73 |
| Mitotic rate (<1mm^2^ vs ≥1mm^2^) | 1.73 (0.37-8.22) | 0.49 | 1.68 (0.86-3.28) | 0.13 | 3.10 (0.96-10.06) | 0.06 |
| Vascular invasion (absent vs present) | 3.28 (1.31-8.20) | 0.01 | 1.71 (0.96-3.05) | 0.07 | 1.91 (0.96-3.82) | 0.07 |
| Preexisting nevi (absent vs present) | 0.28 (0.12-0.65) | 0.003 | 0.73 (0.50-1.07) | 0.11 | 0.52 (0.31-0.87) | 0.01 |
